# Clinicopathological Characteristics With the Status of Mismatch Repair Deficient Invasive Colorectal Cancer With Spontaneous Regression

**DOI:** 10.1002/deo2.70216

**Published:** 2025-10-06

**Authors:** Fumiya Okano, Naohisa Yoshida, Yukiko Morinaga, Naoto Iwai, Reo Kobayashi, Ken Inoue, Osamu Dohi, Takashi Ando, Yoshito Itoh

**Affiliations:** ^1^ Department of Gastroenterology National Hospital Organization Maizuru Medical Center Kyoto Japan; ^2^ Department of Molecular Gastroenterology and Hepatology Graduate School of Medical Science Kyoto Prefectural University of Medicine Kyoto Japan; ^3^ Department of Surgical Pathology Graduate School of Medical Science Kyoto Prefectural University of Medicine Kyoto Japan; ^4^ Ando Clinic Kyoto Japan

**Keywords:** colonoscopy, colorectal cancer, depressive lesion, mismatch repair deficiency, spontaneous regression

## Abstract

Spontaneous regression of endoscopically invasive colorectal cancer (CRC) after biopsy has been rarely reported. We report three cases of endoscopically invasive CRC with spontaneous regression after biopsy and a review of the literature regarding spontaneous regression of CRC with somatic mismatch repair deficiency (MMR‐d). Case 1 involved a 54‐year‐old man who underwent a colonoscopy (CS) after positive fecal immunohistochemical test. A 15‐mm elevated lesion with a depression was detected in the transverse colon, and biopsy results indicated adenocarcinoma. When surgical resection was performed 8 weeks later, the lesion was no longer present. Case 2 involved a 75‐year‐old man with a 10‐mm elevated lesion with a depression in the ascending colon during screening CS. Biopsy results indicated adenocarcinoma. CS was performed 9 weeks later to tattoo the lesion before surgery; however, it was no longer present at that time. Case 3 involved an 84‐year‐old man who underwent surveillance CS after polyp resection and a 12‐mm elevated lesion with a depression was observed in the rectum. Biopsy results indicated adenocarcinoma; therefore, endoscopic resection was scheduled. CS performed 8 weeks later showed the disappearance of the lesion. Mismatch repair deficiency was detected in two of these three patients. The literature search identified 12 cases with the evaluation of MMR, including our three cases, which showed spontaneously regressing colorectal cancer. All 12 lesions had depression; 11 were located on the proximal colon, and 11 cases showed MMR‐d.

## 1 Introduction

Spontaneous regression of any type of carcinoma is rare (frequency of one in 10,000–60,000 cases) [1]. Additionally, spontaneous regression of colorectal cancer (CRC) has a frequency of less than 2% [2]. We report three cases of endoscopically invasive CRC with spontaneous regression after biopsy and a review of the literature regarding spontaneous regression of CRC with somatic mismatch repair (MMR) deficiency (MMR‐d).

## 2 Case Report

### 2.1 Case 1

**FIGURE 1:**
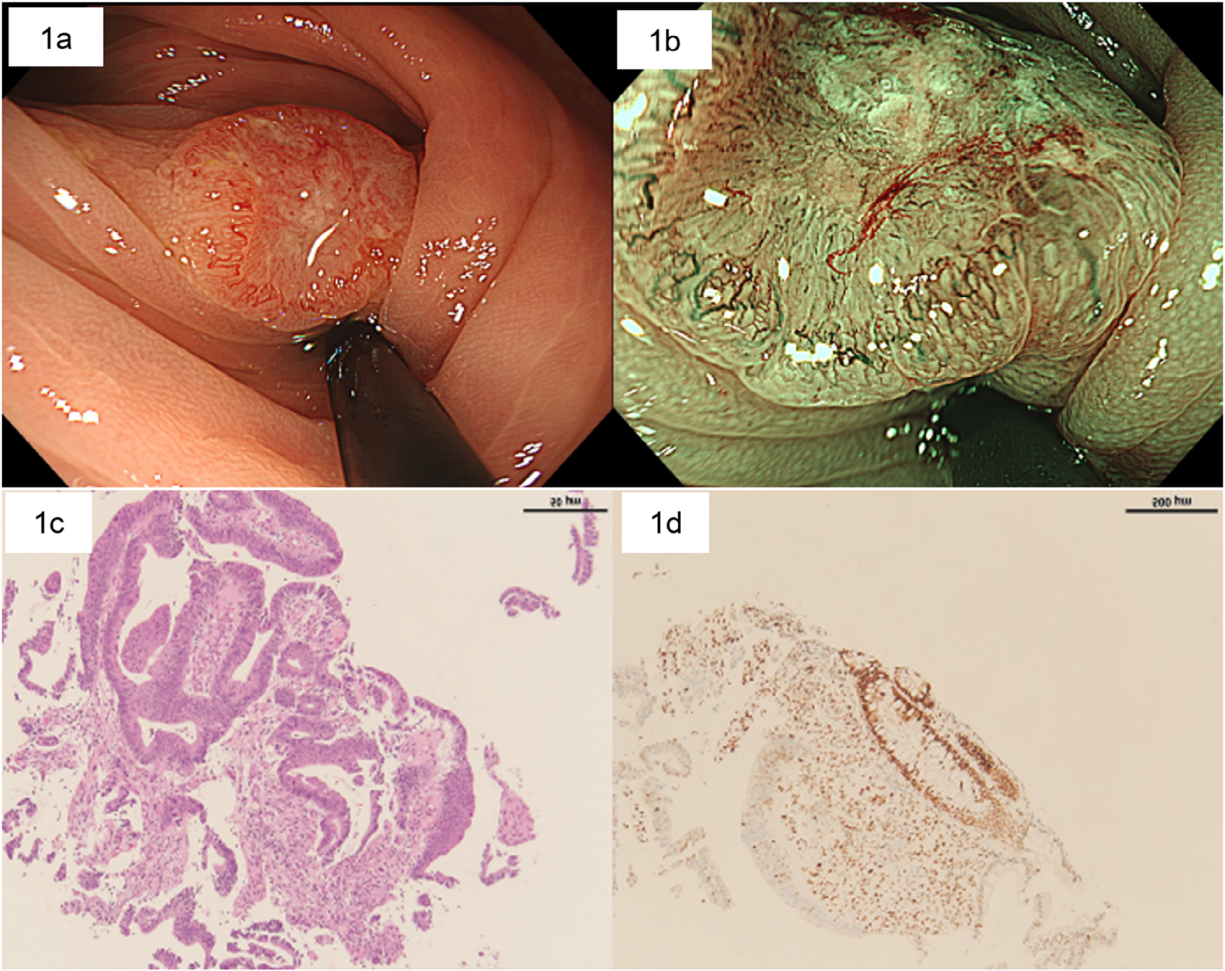
Endoscopic images and histopathology results of Case 1 with spontaneous regression. (a) A 15‐mm elevated lesion with a central depression on the transverse colon (morphology: 0‐IIa+IIc). The surface of the lesion had a slight depression, and rough mucosa was observed. (b) Narrow band imaging (NBI) with magnification shows an amorphous (destroyed) surface pattern. The vessel pattern showed interruption of thick blood vessels. (c) Hematoxylin and eosin staining shows adenocarcinoma. (d) Immunohistochemical staining showed reduced expression of MSH6.

**FIGURE 2:**
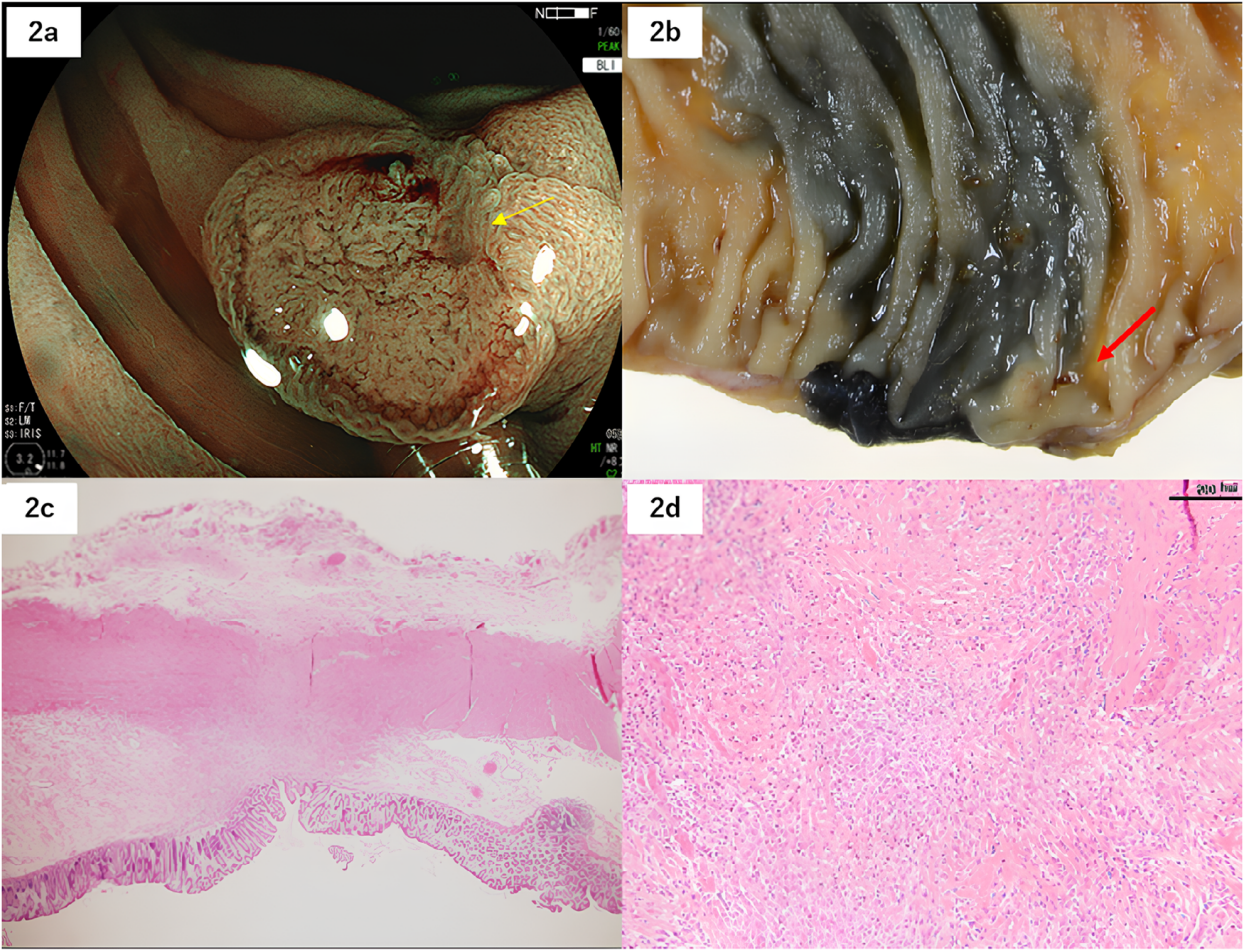
Spontaneous regression Specimen obtained during surgical resection and histopathology results of Case 1 with spontaneous regression. (a) In the endoscopic image of narrow band imaging (NBI) 6 weeks after the biopsy, the lesion size decreased to 10 mm. Biopsy scar (yellow arrow). (b) Only a scar in the excised specimen is observed in the tattooing area (red arrow). (c) Hematoxylin and eosin staining showed a scar without tumor cells. (d) High magnification of the scar showed massive lymphocytic infiltration and fibrosis.

A 54‐year‐old man underwent colonoscopy (CS) at a local clinic because of positive fecal immunohistochemical test. Past history: appendectomy for appendicitis. Medications and supplements: none. During CS, a neoplastic lesion was detected in the transverse colon. Therefore, he was referred to our hospital for further analysis. During a magnifying CS, a 15‐mm superficial elevated lesion with a central depression (morphology: 0‐IIa+IIc) was detected (Figure 1a). Narrow band imaging (NBI) revealed an irregular and amorphous (destroyed) surface pattern and interruption of thick vessels (JNET classification Type 3) (Figure 1b). Chromoendoscopy with crystal violet staining detected a V_I_ pit pattern with high irregularity. T1b cancer was suspected. Subsequent biopsy results indicated well‐differentiated tubular adenocarcinoma (Figure 1c). Immunohistochemical staining for the biopsy specimen showed attenuated expression of MSH6/MSH2 and negative expression of PD‐L1, BRAF, and V600E (Figure 1d). Contrast‐enhanced computed tomography did not show lymph node metastasis or distant metastasis. A clinical diagnosis of cStage1 was determined, and surgical resection was scheduled. A subsequent CS was performed 6 weeks after the initial CS to tattoo the lesion, which was slightly reduced (Figure 2a). During laparoscopic surgery performed 8 weeks after the initial CS, the macroscopic tumor was no longer present, and scarring was observed at the tattooing spot (Figure 2b). A histopathological examination showed no tumor cells in the entire specimen; however, lymphocyte and plasma cell infiltration and mild fibrosis were observed within the fibrotic area of the scar area just below the lamina muscularis mucosa (Figure 2c,d). No recurrence was observed 36 months after the spontaneous regression.

### 2.2 Case 2

**FIGURE 3:**
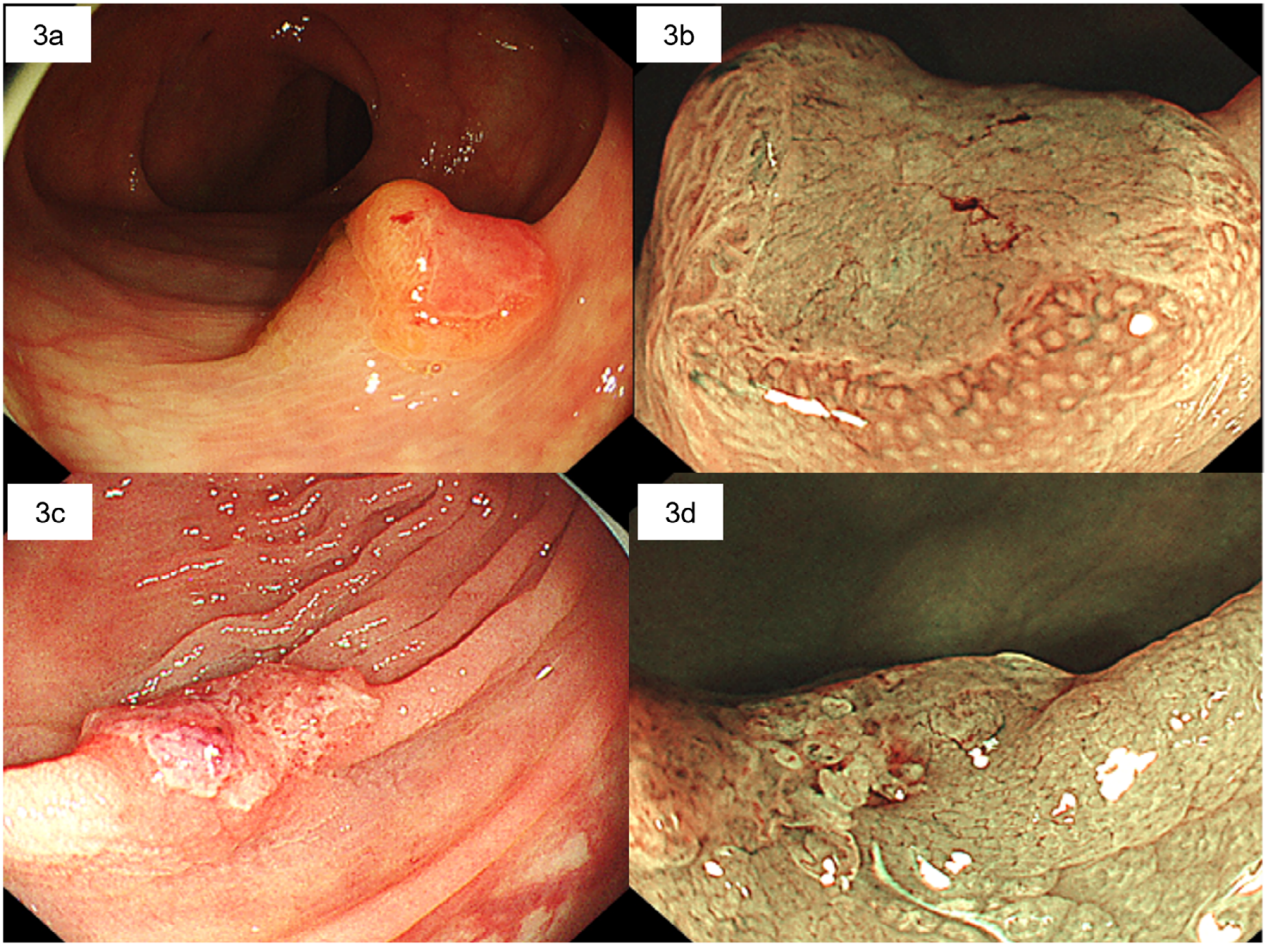
Images of Cases 2 and 3 with spontaneous regression obtained during colonoscopy. (a) A 10‐mm superficial elevated lesion with central depression on the ascending colon (morphology: 0‐IIa+IIc) (Case 2). (b) Narrow band imaging (NBI) with magnification shows an amorphous (destroyed) surface pattern with interuption of thick vessel (Case 2). (c) A 12‐mm superficial elevated lesion with central depression on the rectum (morphology: 0‐IIa+IIc) (Case 3). (d) NBI with magnification showed an amorphous (destroyed) surface pattern (Case 3).

A 75‐year‐old man was referred to our hospital for further examination of a neoplstic lesion observed during a screening CS performed at a local clinic. Past history: aortic dissection. Medications and supplements: sodium risedronate hydrate for osteoporosis, carvedilol for chronic heart failure, an H2 blocker for chronic gastritis, a calcium channel blocker, and an AT1 receptor blocker for hypertension. A magnifying CS detected a 10‐mm superficially elevated lesion with a depression in the ascending colon (morphology: 0‐IIa+IIc). NBI showed a amorphous (destroyed) surface pattern and interruption of thick vessels in the vessel pattern (JNET Type 3)(Figure 3a,b). T1b cancer was suspected. Biopsy results indicated well‐differentiated tubular adenocarcinoma. Immunohistochemical staining of the specimen with MMR showed reduced expression of MLH1/PMS2. Therefore, surgery was scheduled. Endoscopy was performed 9 weeks later to tattoo the lesion; however, the lesion was no longer present, and only scarring was observed. No recurrence was observed 18 months after the spontaneous regression.

### 2.3 Case 3

An 84‐year‐old man underwent a surveillance CS after polyp resection at our center. Past history: appendectomy. Medications and supplements: ursodeoxycholic acid for minor liver injury. A 12‐mm elevated lesion with a depression was detected in the rectum (morphology: 0‐IIa+IIc) (Figure 3c,d). A magnifying CS with NBI detected an amorphous (destroyed) surface pattern (JNET Type 3). T1b cancer was suspected. Biopsy results indicated well‐differentiated tubular adenocarcinoma. Immunohistochemical staining showed no deficiency of MMR. Because of the patient's advanced age, endoscopic resection was scheduled. However, the lesion was no longer present during the CS performed 9 weeks after the initial CS. No recurrence was observed 12 months after the spontaneous regression.

## 3 Discussion

The mechanism of spontaneous regression of CRC has not yet been established; however, various factors have been discussed. Recently, an association between spontaneous regression of CRC and MMR‐d has been reported [3]. The MMR genes include *MLH1*, *MSH2*, *MSH6*, and *PMS2*, and the proteins can form MLH1/PMS2 and MSH2/MSH6 heterodimers. These heterodimers recognize base pair mismatches and losses that occur during DNA replication and repair them. MMR‐d, derived from mutations in these genes, results in defects of the repair system. MMR‐d can be examined with polymerase chain reaction to identify repeat sequences and immunohistochemical staining to identify four MMR proteins (MLH1, MSH2, MSH6, and PMS2). In Japan, the frequency of MMR‐d in CRC was estimated to be 5.9%, which was lower than that in Western countries (13%) [4]. MMR‐d is characterized by marked lymphocytic infiltration surrounding the tumor. Utsumi et al. suggested that somatic MMR‐d in the tumor induces an immune response of T cells to neoantigens expressed in cancer cells, resulting in spontaneous tumor regression [5, 6]. Harata et al. also reported that such immune responses are strongly triggered by stimulation, such as that associated with biopsies [3]. Koen et al. reported a patient with metastatic liver tumors associated with CRC who underwent biopsy and experienced spontaneous liver tumor regression [7]. MMR‐d of the biopsy specimen was confirmed for that case, suggesting that the biopsy might trigger spontaneous lesion regression [7]. According to these reports, in our cases with MMR‐d, the immune response of T cells seemed to be strong and enhanced by biopsy. These probably induced marked lymphocytic infiltration, resulting in lesion regression.

In the current report, in Case 1, MMR‐d and marked lymphocytic infiltration in the area where cancer had been observed were noted, suggesting that the biopsy triggered an immune response related to MMR‐d. However, the CD4 and CD8 lymphocyte counts were not biased. In Case 2, MMR‐d was also confirmed, suggesting an immune response attributable to the biopsy. In Case 3, MMR‐d was not observed. However, the biopsy specimen showed marked lymphocytic infiltration, and an immune response was suspected. In these three cases, it was not examined which genes referred to MMR‐d.

A search of the literature in English published between 2000 and 2023 yielded 14 case reports of spontaneous regression of CRC. We reviewed 12 of those cases with somatic MMR‐d as well as our three cases (Table 1) [8, 9, 10]. The mean age of the 12 patients was 71.2 years (standard deviation, ±8.3 years), and 6.7% (eight patients) of those patients were male. The location of 91.7% (11 cases) of the lesions was the proximal colon, and all lesions had a depressed area. Although the morphologic type of one case in the literature (Case 4 in Table 1) was considered Is, we found a slight depression within the elevation. Therefore, it was diagnosed as a depressed lesion (morphology: Is+IIc). Regarding the estimated depth, eight reached the submucosal layer and four reached the proper muscle layer. The time between the biopsy and regression was ≤9 months for 75% (nine cases) of the cases.

**TABLE 1:** Colorectal cancer (CRC) with spontaneous regression cases with mismatch repair deficiency (MMR) status.

Case	Author	Age	Sex	Location in the colon and rectum	Macroscopic type	Histopathology	The estimated depth of invasion	Treatment	Duration of regression after biopsy(week)	Follow‐up (year)	The estimated cause of regression	Immunohistochemistry finding: mismatch repair protein lost
1	Karakuchi	78	M	T	Type 2	Por	MP	Surgery	8	1	Biopsy	MLH1/PMS2
2	Nishiura	67	F	T	Type 2	Por	MP	Surgery	21	5	Immune response	MLH1/PMS2
3	Yokota	76	F	T	Is+IIｃ	Mod	SM	Surgery	7	5.5	Biopsy, immune response	MLH1/PMS2
4	Yokota	64	F	C	*Is+IIc	Well	SM	Surgery	12	5	Biopsy, immune response	MSH2/MSH6
5	Yokota	64	M	T	Type 2	Mod	MP	Surgery	6	6	Biopsy, immune response	MLH1/PMS2
6	Harata	76	F	T	Type 2	Well	MP	Surgery	8	6	Biopsy, immune response	MLH1/PMS2
7	Utsumi	78	M	A	IIa+IIc	Well	SM	Surgery	5	1.5	Biopsy, immune response	PMS2
8	Utsumi	66	M	A	IIa+IIc	Mod	SM	Surgery	7	1.5	Biopsy, immune response	MLH1/PMS2
9	Utsumi	73	M	A	IIa+IIc	Mod	SM	Surgery	15	1.5	Biopsy, immune response	MLH1/PMS2
10	Case 1	54	M	T	IIa+IIc	Well	SM	Surgery	8	3	Biopsy, immune response	MSH2/MSH6
11	Case 2	75	M	A	IIa+IIc	Mod	SM	Nothing	9	1.5	Biopsy, immune response	MLH1/PMS2
12	Case 3	84	M	R	IIa+IIc	Well	SM	Nothing	8	1	Biopsy, immune response	No deficiency

Abbreviations: A, ascending colon; CRC, colorectal cancer; F, female; M, male; MMR, mismatch repair; Mod, moderately differentiated tubular adenocarcinoma; MP, muscularis propria; Por, poorly differentiated adenocarcinoma; R, rectum; SM, submucosa; T, transverse colon; Well, differentiated tubular adenocarcinoma, well differentiated.

The specific treatment required for spontaneous regression of CRC has not yet been determined. Surgery is recommended because of the possibility of remaining microscopic tumor cells. However, our patients in Cases 2 and 3 did not undergo surgery, and lesion relapse did not occur during careful follow‐up. Therefore, especially in elderly patients or those at high surgical risk, close surveillance could also be considered an alternative management option. Further studies and cases are necessary to clarify the mechanism of spontaneous regression and the treatment plan for cases of spontaneous regression of CR. Based on our findings, when a CRC that spontaneously regresses after biopsy is identified, it is desirable to perform testing for MMR‐d and to accumulate such cases and related data.

There were several limitations in this case report. The tumor depth of each lesion was diagnosed endoscopically because of spontaneous regression. Methylation of MLH1 was not examined, though methylation of MLH1 is related to right‐sided CRC with MMR‐d in elderly people like our cases. We did not analyze EPCAM, though it is related to MMR‐d.

## Author Contributions

**Fumiya Okano** and **Naohisa Yoshida** contributed to the study conception and design. Data collection and analysis were performed by **Fumiya Okano**, **Naohisa Yoshida**, **Reo Kobayashi**, **Ken Inoue**, **Osamu Dohi**, and **Takashi Ando**. **Yoshito Itoh** helped to design this study. Histopathological assessment was performed by **Yukiko Morinaga**. The first draft of the manuscript was written by **Fumiya Okano** and **Naohisa Yoshida**, and all authors read and approved the final manuscript.

## Conflicts of Interest

The authors declare no conflicts of interest.

## Ethics Statement

**Human/Animal Rights**: All procedures followed were in accordance with the ethical standards of the responsible committee on human experimentation (institutional and national) and with the Helsinki Declaration of 1975, as revised in 2008. This study was approved by the ethics committee of Kyoto Prefectural University of Medicine (ERB‐C‐1600; approval data: December 23, 2019) as a partial study of our large‐scale retrospective and prospective study.

## References

[1] H. E. Kaiser , B. Bodey, Jr , S. E. Siegel , et al., “Spontaneous Neoplastic Regression: The Significance of Apoptosis,” In Vivo 14 (2000): 773–788. 11212857

[2] A. S. Abdelrazeq , “Spontaneous Regression of Colorectal Cancer: A Review of Cases From 1900 to 2005,” International Journal of Colorectal Disease 22 (2007): 727–736. 17146588 10.1007/s00384-006-0245-z

[3] S. Harata , H. Takahashi , N. Ando , et al., “Spontaneous Regression of Advanced Transverse Colon Cancer With Deficient Mismatch Repair: A Case Report,” Surgical Case Reports 9 (2023): 64. 37095273 10.1186/s40792-023-01595-x PMC10126167

[4] S. Asaka , Y. Arai , Y. Nishimura , et al., “Microsatellite Instability‐low Colorectal Cancer Acquires a KRAS Mutation During the Progression From Dukes' A to Dukes' B,” Carcinogenesis 30 (2009): 494–499. 19147861 10.1093/carcin/bgp017

[5] K. Chida , K. Nakanishi , H. Shomura , et al., “Spontaneous Regression of Transverse Colon Cancer: A Case Report,” Surgical Case Reports 3 (2017): 65. 28488173 10.1186/s40792-017-0341-z PMC5423878

[6] T. Utsumi , S. Miyamoto , T. Shimizu , et al., “Spontaneous Regression of Mismatch Repair‐deficient Colorectal Cancers: A Case Series,” Digestive Endoscopy 33 (2021): 190–194. 32416608 10.1111/den.13723

[7] Z. Koen , R. Dieuwertje , G. Magda , et al., “Spontaneous Complete Regression of Colon Cancer Liver Metastases in a Lung Transplant Patient: A Case Report,” Case Reports in Transplantation 2023 (2023): 9643370. 36685719 10.1155/2023/9643370 PMC9851788

[8] N. Karakuchi , M. Shimomura , K. Toyota , et al., “Spontaneous Regression of Transverse Colon Cancer With High‐frequency Microsatellite Instability: A Case Report and Literature Review,” World Journal of Surgical Oncology 17 (2019): 19. 30646898 10.1186/s12957-018-1552-x PMC6334436

[9] B. Nishiura , K. Kumamoto , S. Akamoto , et al., “Spontaneous Regression of Advanced Transverse Colon Cancer With Remaining Lymph Node Metastasis,” Surgical Case Reports 6 (2020): 100. 32394212 10.1186/s40792-020-00858-1 PMC7214569

[10] T. Yokota , Y. Saito , H. Takamaru , et al., “Spontaneous Regression of Mismatch Repair‐Deficient Colon Cancer: A Case Series,” Clinical Gastroenterology and Hepatology 19 (2021): 1720–1722. 32858199 10.1016/j.cgh.2020.08.051

